# Effect of Kelp Waste Extracts on the Growth and Development of Pakchoi (*Brassica chinensis* L.)

**DOI:** 10.1038/srep38683

**Published:** 2016-12-09

**Authors:** Shiyan Zheng, Jie Jiang, Meilin He, Shanmei Zou, Changhai Wang

**Affiliations:** 1 Jiangsu Provincial Key Laboratory of Marine Biology, College of Resources and Environmental Sciences, Nanjing Agricultural University, Nanjing 210095, China

## Abstract

To explore the effects of kelp waste extracts (KWE) on the growth and development of *Brassia chinensis* L., germination and greenhouse experiments were carried out under different concentrations of KWE. The results showed that a higher germination percentage (95%), associated with high germination index (8.70), germination energy (71.67%) and seedling vigor index (734.67), was obtained under a lower KWE concentration (2%) compared with the control. The radicle length (4.97 cm), fresh weight (0.32 g/10 seedlings) and dry weight (0.015 g/10 seedlings) were significantly increased in the treatment of 2% KWE. KWE also could enhance the root growth, the maximum leaf length × width and the fresh weight of plants, the optimal value of which increased by 8.37 cm, 58.14 cm^2^ and 7.76 g under the treatment of 10% KWE compared with the control respectively. Meanwhile, the contents of vitamin C and soluble sugars in pakchoi leaf were improved by 19.6 mg/100 g and 1.44 mg/g compared with the control, and the nitrate content was decreased by 212.27 mg/kg. Briefly, KWE could markedly stimulate the pakchoi seeds germination at a lower concentration (2%) and enhance the plant growth and quality at a higher concentration (10%).

As biostimulants and liquid fertilizers, seaweed extracts have been applied as foliar spray and soil drench in organic farming^1^. Unlike chemical fertilizers, seaweed extracts are biodegradable, non-polluting, non-toxic and non-hazardous to humans and animals^2^. Thus, natural seaweed extracts has been allowed to partially replace the conventional synthetic fertilizer^3^,^4^. The bioeffects of natural seaweed extracts have been explored on some vegetable, fruit, flower and ornamental crops as well as turf grasses^5^. With the wide use of seaweed extracts in agriculture, many positive effects were reported, such as stimulated seed germination, enhanced plant growth, root development, leaf quality and yield, improved resistance to biotic and abiotic stresses, as well as increased post harvest shelf life^3^,^6^. Besides, the contents of total soluble protein, phenolics and flavonoid, as well as antioxidant capacity of the plants could be increased under the treatment of seaweed extracts^7^. Battacharyya *et al*.^5^ illustrated diverse seaweed extracts regulated the gene expression of nutrient uptake in treated plants. These effects have been attributed to the presence of phytohormones, quaternary ammonium molecules such as proline and betaines, related larger molecules such as unique polysaccharides and polyphenols, or mineral elements in seaweed extracts^6^,^8^.

Recent research mostly focused on obtaining seaweed extracts from fresh seaweeds, rather than the much cheaper seaweed waste^9^. Kelp waste is the residue after extracting alginate from kelp (*Laminaria japonica* Aresch.). Due to the worldwide intensive requirements for alginate production and the low extracting efficiency, a large amount of kelp wastes are being produced each year in China^10^. These wastes contain massive crude fiber, protein as well as residual alginic acid. The degradation of these substances could generate a mass of organic nutrients and nutrient salts, which could stimulate the growth of plant or microbial^11^. Currently, most of these solid wastes are directly released with the landfill, which results in serious waste of resources and occupation of much land^12^. Hence, the reuse of kelp waste has great promise in alleviating waste pollution and developing organic and sustainable agriculture.

The effect of fresh seaweed extracts on plant has been investigated, but there are few reports on the impact of seaweed waste extracts on agricultural crops. Pakchoi (*Brassia chinensis* L.) is one of the most important vegetables in China^13^. With the rapid growth of population, the simultaneous enhancement on production and quality of pakchoi is the main problem in organic agriculture. The aims of this research are to probe the effects of kelp waste extracts (KWE) on the germination, growth, yield and quality of pakchoi (*Brassia chinensis* L.) and to explore the potential of KWE as biostimulants and biofertilizers on agriculture.

## Results

### Physiochemical characteristics of kelp waste and KWE

Kelp waste contains various organic substances and mineral nutrients. The composition analysis of kelp waste showed that the organic substances were very rich (Table 1). The nitrogen (N) concentration was higher than other mineral elements followed by calcium (Ca). After extracting with enzymolysis, KWE was also rich in soluble sugars, amino acids as well as diverse mineral elements with high concentrations of N, phosphorus (P), potassium (K), Ca, and magnesium (Mg)^14^. The above data showed that both kelp waste and KWE may be considered as versatile biostimulants to provide sufficient nutrients for the growth and development of plants.

### Effect of KWE on seeds germination and pakchoi seedlings growth under laboratory conditions

**Figure 1:**
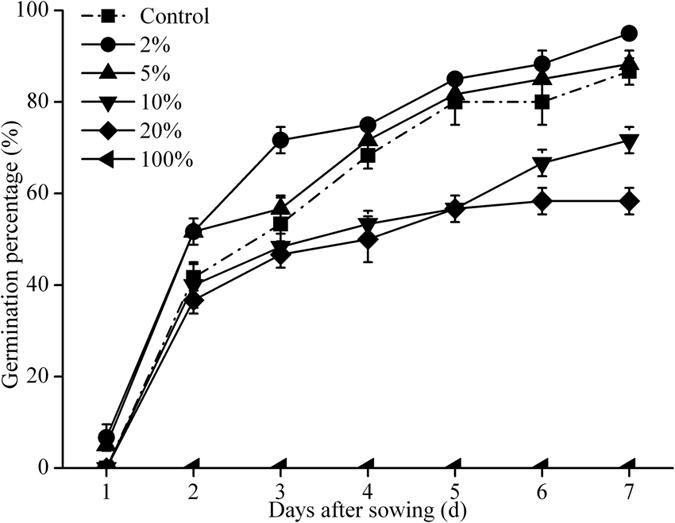
Effects of KWE on the germination percentage of pakchoi seeds. Values are presented as mean ± SD (n = 100 seeds).

The lower concentration of KWE (2%) could significant stimulate the germination of pakchoi seeds. As shown in Fig. 1, the pakchoi seeds soaked with 2–5% of KWE presented higher germination rates, while higher concentrations of KWE (10–100%) inhibited germination, no seeds were observed to germinate in the treatment of 100% KWE. The highest GP (95%) was obtained under the treatment of 2% KWE, which increased by 8.33% compared with the control (Table 2). Similar trends in the parameters of GI, GE and SVI were observed, which increased by 2.35, 18.34% and 273.97 at the treatment of 2% KWE compared with the control respectively (Table 2).

**Figure 2:**
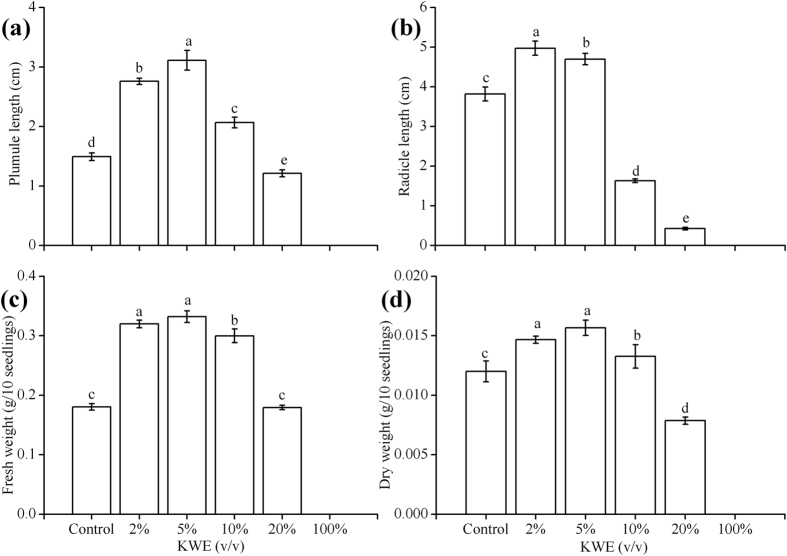
Effects of KWE on pumule length (**a**), radicle length (**b**), fresh weight (**c**), and dry weight (**d**) of pakchoi seedlings. Values are mean ± SD (n = 50 seedlings). Different letters above the bars indicate the differences are significant at *p* < 0.05.

The lower concentrations of KWE (2–5%) could effectively improve the growth performance of pakchoi seedlings. The plumule length, radicle length, fresh weight and dry weight of pakchoi seedlings were significantly enhanced by the treatment of KWE with a concentration lower than 5% (Fig. 2). The maximal plumule length (3.11 cm, Fig. 2a), fresh weight (0.33 g/10 seedlings, Fig. 2c) and dry weight (0.016 g/10 seedlings, Fig. 2d) were increased by 1.62 cm, 0.15 g and 0.0037 g under the treatment of 5% KWE compared with the control, respectively. The optimal value of the radicle length (4.97 cm) was obtained in the treatment of 2% KWE, which increased by 1.15 cm compared with the control.

### Effect of KWE on pakchoi growth in greenhouse

**Figure 3:**
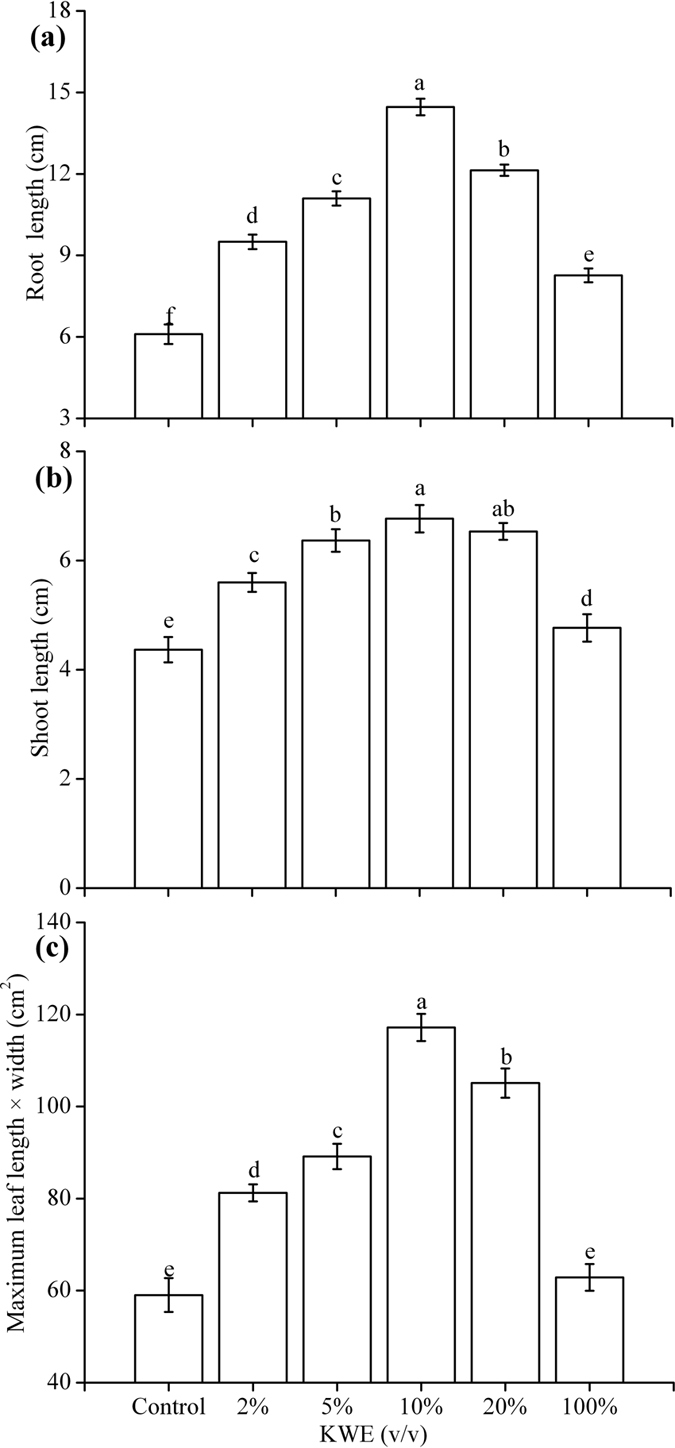
Effects of KWE on root length (**a**), shoot length (**b**) and maximum leaf length × width (**c**) of pakchoi plants under greenhouse. Values are mean ± SD (n = 9 plants). Different letters above the bars indicate the differences are significant at *p* < 0.05.

In the greenhouse experiment, KWE showed obvious improvement on the growth of pakchoi plants (Fig. 3). All KWE treatments displayed significant increases in root length (Fig. 3a) and shoot length (Fig. 3b) compared with the control (*p* < 0.05). Except the treatment with 100% KWE, the maximum leaf length × width (Fig. 3c) in treatments with 2–20% KWE was markedly higher than the control (*p* < 0.05). The treatment of 10% KWE showed the highest values of shoot length (6.77 cm), root length (14.47 cm) and maximum leaf length × width (117.18 cm^2^), which increased by 2.40, 8.37 cm and 58.14 cm^2^ compared with the control, respectively. The treatment with 10% KWE was the optimal concentration for stimulating pakchoi plants growth, while high concentration of KWE (100%) displayed non-significant effect in growth-promotion.

**Figure 4:**
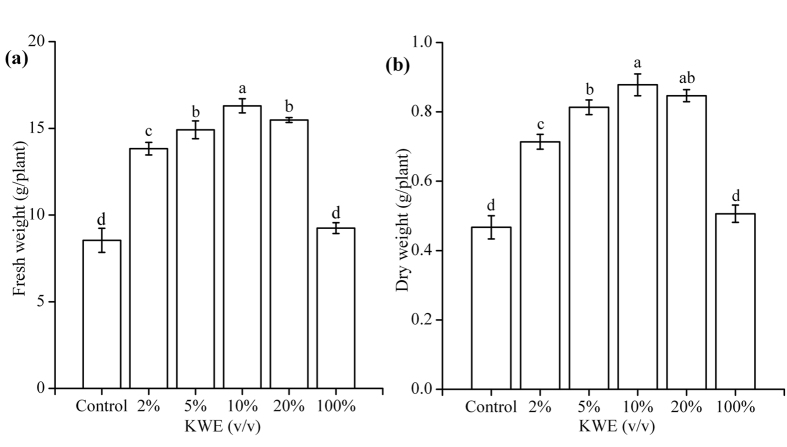
Effects of KWE on fresh weight (**a**) and dry weight (**b**) of pakchoi plants in the greenhouse. Values are mean ± SD (n = 9 plants). Different letters above the bars indicate the differences are significant at *p* < 0.05.

Positive effects on the biomass of pakchoi plants were observed with application of KWE as foliar spray (Fig. 4). Significant increases in fresh weight (Fig. 4a) and dry weight (Fig. 4b) were obtained under the treatments of 2–20% KWE (*p* < 0.05). The treatment with 10% KWE showed the highest fresh weight (16.30 g/plant) and dry weight (0.88 g/plant), which separately increased by 7.76 and 0.41 g compared with the control.

### Effect of KWE on pakchoi quality in greenhouse

**Figure 5:**
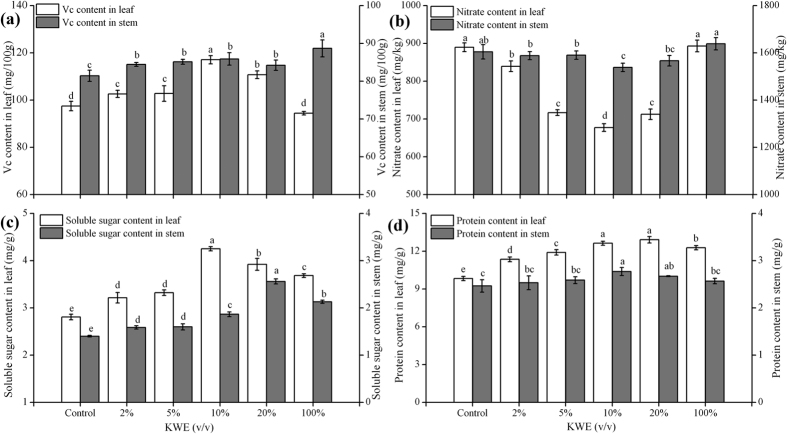
Effects of KWE on vitamin C (**a**), nitrate (**b**), soluble sugar (**c**) and soluble protein (**d**) contents in pakchoi plants under greenhouse. Values are mean ± SD (n = 9 plants). Different letters above the bars indicate the differences are significant at *p* < 0.05.

The treatments with 2–20% KWE as foliar spray application proved to improve the quality of pakchoi plants. The leaf and stem of pakchoi plants exhibited different responses to KWE, as in the contents of vitamin C (Vc), nitrate, soluble sugars and soluble protein (Fig. 5). The highest Vc (117.09 mg/100 g, Fig. 5a) and soluble sugar contents (4.25 mg/g, Fig. 5c) in pakchoi leaf were gained in the treatment of 10% KWE, which increased by 19.60 mg/100 g and 1.44 mg/g compared with the control. However, the maximal contents of Vc (88.70 mg/100 g) and soluble sugar (2.56 mg/g) in pakchoi stem were achieved in 100% KWE treatment and 20% KWE, respectively. The soluble protein content in pakchoi leaf reached the highest under the treatment with 20% KWE (12.92 mg/g, Fig. 5d), while that in pakchoi stem peaked under the treatment of 10% KWE (2.77 mg/g). With regard to the dynamic of nitrate contents in treated plants, the nitrate content in pakchoi leaf was almost lower than that of pakchoi stem (Fig. 5b). The treatments of 2–20% KWE could debase the nitrate content in pakchoi plants. The minimum nitrate contents of pakchoi leaf and stem were obtained under 10% KWE treatment, which reduced by 212.27 and 66.04 mg/kg compared with the control (Fig. 5b). There was no decrease in nitrate content of pakchoi plants treated with 100% KWE.

**Figure 6:**
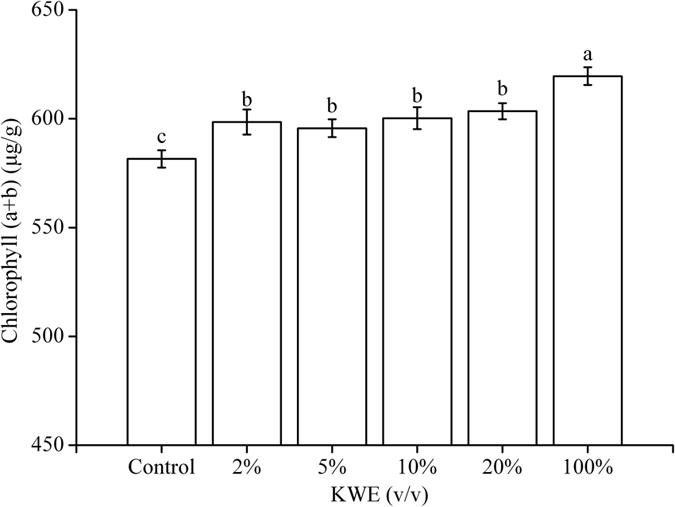
Effect of KWE on chlorophyll (a + b) content in pakchoi plants in greenhouse. Values are mean ± SD (n = 9 plants). Different letters above the bars indicate the differences are significant at *p* < 0.05.

As shown in Fig. 6, KWE significantly increased the chlorophyll (a + b) content in treated pakchoi plants. However, the treatments with 2–20% KWE elicited no obvious differences in chlorophyll (a + b) content of pakchoi leaf (*p* < 0.05). The highest chlorophyll (a + b) content (619.57 μg/g) in pakchoi leaf was observed under 100% KWE treatment, which increased by 38.01 ug/g compared with the control. The chlorophyll (a + b) content in pakchoi leaf treated with 2–20% KWE was increased by 14.08 to 21.89 ug/g compared to the control.

## Discussion

Pakchoi seeds treated with low concentrations of KWE (2–5%) responded better in germination rate associated with higher GI, GE and SVI, as well as higher plumule length, radicle length and biomass. However, higher concentrations of KWE (10–100%) exhibited significant inhibition on the germination and seedlings growth of pakchoi, especially 100% KWE treatment. This may be attributed to the fact that most plants have lower tolerance to salinity during germination and seedling growth^15^. The total salinity of 100% KWE was 8.34 ± 0.14 g/L, and the content of Na^+^ ion reached 5.22 ± 0.07 g/L in this research. The high concentration accumulations of Na^+^ and Cl^−^ ions might restrain the embryo or the seedlings development of many vegetable crops, resulting in the inhibition on germination, uneven morphogenesis and the decline of crop production^16^.

The stimulated germination rate of pakchoi seeds under lower concentrations of KWE might result from combined effects induced by the presence of soluble sugars, amino acids and various mineral elements in KWE^17^. Plants have different tolerance to nutrients in different growth stage. The improved growth performance in treated pakchoi plants under higher concentrations of KWE (10–20%) may be also attributed to the presence of these nutrient substances in KWE. These constituents could improve the growth performance and yield of agricultural crops through affecting cellular metabolism. For example, the organic substances in KWE could induce strong physiological responses^18^. Considering that sugars act both as effective signaling molecules and as immediate substrates for intermediary metabolism, availability of sugars is a powerful driver for plant growth and development^19^,^20^. Soluble sugars also could enhance plant growth similar to hormones^21^. Some amino acids such as proline were osmoprotectants, which had great importance in improved stress tolerance of plant^6^,^22^. The growth rate of plant is closely related to N supply^23^. Alginic acid displays soil-conditioning properties and can chelate metal ions, which markedly stimulates root growth by directly or indirectly associated with soil microbes^5^,^8^. Hernández-Herrera *et al*.^17^ found phosphorous in seaweed extracts facilitates root proliferation increasing the root/shoot ratio. The K in seaweed extracts has a positive effect on enhancing photosynthesis and meristematic growth and regulating water status in treated plants^17^. The Ca present in KWE facilitates improving cell elongation, cell stability and enzyme activation in plants. Besides, it has been confirmed that foliar application of seaweed extracts enhances root development in varieties of crops^6^. This research illustrated that the use of kelp waste extracts dramatically stimulated root growth of pakchoi (Fig. 3a). The enhancement on root growth may improve the growth and yields of aboveground plants^3^. This might account for the increase of biomass in pakchoi plants treated with KWE.

Recent research^3^,^6^,^7^ suggested natural seaweed extracts have great influence on plant metabolism, such as increasing antioxidant ability, total soluble protein and phenolics content, etc. The metabolisms of carbon, nitrogen and sulfur as well as photosynthesis were significantly induced by the seaweed extracts^24^. In this research, the contents of Vc, soluble sugars, soluble protein and chlorophylls in treated plants were markedly enhanced by the KWE (Figs 5 and 6), the maximum of which were increased by 19.60 mg/100 g, 1.44 mg/g, 3.08 mg/g and 38.01 μg/g in comparison with the control, respectively. Meanwhile, the minimum nitrate content in pakchoi plants was obtained under 10% KWE treatment, which was reduced by 212.27 mg/kg compared with the control. This might be attributed that seaweed extracts through up-regulating the expression of nitrate transporter gene stimulated nitrogen sensing, improving nitrogen assimilation^5^,^25^. Sugars in KWE are indispensable to provide energy and carbon building blocks for protein and RNA biosynthesis in plant^26^. An adequate supply of free amino acids is essential for meeting any changes in growth rates and protein synthesis under normal growth conditions^23^. It has been confirmed that free amino acids could regulate the uptake of NO_3_^−^ and NH_4_^+ ^23. The exogenous supply of amino acids could reduce the uptake of NO_3_^−^ in *Arabidopsis thaliana*^27^. The containing mineral elements also facilitate increasing the total soluble protein content in treated plants^28^. In addition, the increased chlorophylls in treated plants indicate that application of KWE could decrease chlorophylls degradation and delay plant senescence^6^, which was in accordance with the related results^7^,^24^. The above physiological parameters are the indicators to assess the pakchoi quality. Thus, this research implies that the improvement on pakchoi quality can be obtained under the treatment of extracts from kelp waste. The molecules exogenously added onto the plant leaves are actively taken up and go into metabolism. It was confirmed that hexoses, disaccharides and various amino acids are readily absorbed when exogenously applied onto leaves^29^. Besides, Sultana *et al*.^30^ pointed out that foliar spray of calcium nitrate (Ca(NO_3_)_2_), manganese sulfate (MnSO_4_) and dipotassium phosphate (K_2_HPO_4_) could partially alleviate the negative impacts of salinity on photosynthesis, dry matter accumulation and yield through mitigating the nutrient requirements of salt-stressed plants. And the nutrients were mainly absorbed by the leaves when applied onto the shoot. Therefore, further work for elucidating the complicated mechanism of KWE on pakchoi growth and development needs to be done.

Any improvements in agriculture should decrease the adverse environmental effects and enhance the sustainable development of agricultural system^31^. Seaweeds are one of important marine renewable resources, which have been widely used as food, biofertilizer, feed, and sources of various fine chemicals such as agar, alginate and carrageenan^32^. Considering the direct or indirect stimulatory impacts on plant metabolism, seaweed extracts have been worldwide used as plant biostimulants^9^. The results in this experiment, which elicited that the bioeffects of extracts from kelp waste on the seeds germination and the growth of pakchoi, agreed with the recent studies related to fresh seaweed extracts^5^,^28^,^31^. The application of KWE as seeds soaking and foliar spray was effective in stimulating seeds germination and establishment, enhancing plant growth as well as improving plant quality. Moreover, KWE contains substantial amounts of plant growth-promoting compounds, the production cost of which has been conducted in previous study^14^. The feedstock for KWE production is very cheap, even free. KWE is biodegradable and eco-friendly to the environment. Thus, KWE could be considered as an alternative source for synthesizing plant growth regulators, biostimulants or biofertilizers, which will have a great potential for the enhancement on yield and quality in organic agriculture. The action and application modes of seaweed extracts on various crops under different conditions were considerably complex^9^. It needs further work to probe the bioeffect and mechanism of KWE with different application patterns on diverse crop growth and stress tolerance, which will be greatly significant in promoting the use of KWE in organic agriculture.

## Methods

### Preparation of kelp waste extracts

Kelp waste was kindly provided by Shandong Jiejing Group Co., Ltd. in China. The preparation and composition analysis of kelp waste extracts (KWE) were performed as previously described by Zheng *et al*.^14^. Kelp waste powder with the diameter less than 0.50 mm was suspended into 0.2 M phosphate buffer solution (PBS) and hydrolyzed with cellulase, pectinase and papain sequentially. The hydrolysate was collected by centrifugation at 6800 g for 10 min. The supernatant was KWE, which was stored at −20 °C until use.

### Bioassay for germination test

Pakchoi seeds were purchased from Nanjing Ideal Agricultural Science and Technology Co., Ltd. in China. Germination was recorded daily over a period of 7 days. A total of 6 different treatments were conducted for this experiment, and 5 groups of 20 seeds were tested for germination per treatment. Before treatment with KWE, pakchoi seeds were surface-sterilized with 4.0% sodium hypochlorite solution for 15 min at room temperature and subsequently triple-rinsed with sterile distilled water. Tested pakchoi seeds were placed on filter papers (Double Ring 102) in sterilized 90 mm Petri dishes, and then treated with 5 mL of different concentrations of KWE (2, 5, 10, 20, and 100%) and distilled water (control). The plates were incubated at 25 ± 1 °C in a light/dark (16/8 h) period with a light intensity of 120 μmol/m^2^/s. When the radicle had protruded more than 2 mm, germination was considered to have occurred. The variables such as germination parameters, radicle length, plumule length, fresh weight and dry weight of pakchoi seedlings were measured. Germination percentage (GP), germination index (GI), germination energy (GE) and seedling vigor index (SVI) were calculated according to the methods described by Hernández-Herrera *et al*.^17^.

### Greenhouse growth bioassay

The treatments in greenhouse experiment were similar to germination test. Two hundred and sixteen 7-day-old seedlings were selected and randomly distributed to different treatment groups. A total of 6 different treatments were performed with each of the 36 replications. Pakchoi seedlings were transplanted into polyethylene plastic pots (100 mm diameter × 80 mm height) containing 400 g fresh garden soil (N: P_2_O_5_: K_2_O = 0.1397: 0.0824: 0.0572 g/kg) and grown in a growth chamber at 28/22 °C (day/night) in light/dark (16/8 h) period. Plants were treated with 60 mL different dilutions of KWE (2.0, 5.0, 10, 20, and 100%) as foliar spray for 7 days after transplanting and then every 5 days for 3 times in total. Water-treated plants were used as the control. Potted plants were grown for 30 days. Morphological characteristics such as root length, shoot length, the maximum leaf length and leaf width, fresh weight and dry weight were recorded.

### Analysis of pakchoi quality in greenhouse

To explore KWE on the quality of pakchoi, the contents of Vc, nitrate, soluble sugars, soluble protein and chlorophylls in plants were analyzed in this research. A total of 9 plants were collected in each treatment to determine the above parameters. The contents of Vc in the stem and leaf of plants were separately assessed using the Indophenol Xylene method^33^. The contents of nitrate in the leaf and stem were separately determined using the Salicylic Acid-Sulfuric Acid colorimetry^34^.

Soluble sugar contents in the stem and leaf of plants were individually determined by a modified Phenol-Sulfuric Acid method^35^. Before determining, all samples were under heat-treatments. A sample of 0.1–0.3 g leaf or shoot was cut into pieces, placed into a 25 mL graduated test tube, and then 10 mL pure water was added. The tube was then heated at 100 °C for 30 min in a water bath. The sample solution was transferred to a 25 mL volumetric flask and made up to 25 mL with distilled water. The above solution was centrifuged at 4 °C for 10 min. Then, 1 mL supernatant was mixed with 0.5 mL of 6.0% (v/v) phenol solution and 2.5 mL concentrated sulfuric acid. The mixture was further heated at 45 °C for 30 min in a water bath, and then cooled to room temperature. The absorbance at 490 nm was determined by SpectraMax M5 Microplate Reader (Molecular Device, US).

Soluble protein contents in the stem and leaf of plants were separately estimated according to the Coomassie Brilliant Blue method^36^. Each sample of 0.5 g fresh material was weighed and macerated in 5 mL of 0.1 M PBS (pH 7.4). The homogenate was centrifuged at 4 °C, 6800 g for 10 min. Then, 1 mL of the supernatant was transferred into a 10 mL test tube and 5 mL of 0.1 g/L coomassie brilliant blue G-250 solution was added. The mixture was shaken well and allowed to stand for 5 min at room temperature. The absorbance of the sample at 595 nm was measured using SpectraMax M5 Microplate Reader. A standard curve of protein was prepared using bovine serum albumin to calculate the protein content in the samples.

The chlorophyll content in the leaf of plants was measured according to the method described by Lichtenthaler & Wellburn^37^. Each sample of 0.5 g leaf was cut into pieces and placed into a 50 mL volumetric flask. Then 50 mL of 100% methanol was added into the flask and extracted at 4 °C for 24 h in darkness. The absorbance of methanol-extracts at 653 and 666 nm was determined with SpectraMax M5 Microplate Reader.

### Statistical analysis

All data was expressed as mean ± standard deviation (SD) and analyzed using one-way analysis of variance (ANOVA) with Least Significant Difference (LSD) test at a 5% level. All statistical analyses were performed with SPSS version16.0 (SPSS Inc., Chicago, IL, USA). All plots were created using Origin 8.5 (OriginLab Corporation, Northampton, MA).

## Additional Information

**How to cite this article**: Zheng, S. *et al*. Effect of Kelp Waste Extracts on the Growth and Development of Pakchoi (*Brassica chinensis* L.). *Sci. Rep.* **6**, 38683; doi: 10.1038/srep38683 (2016).

**Publisher's note:** Springer Nature remains neutral with regard to jurisdictional claims in published maps and institutional affiliations.

## Figures and Tables

**Table 1:** The main composition of kelp waste.

Organic substances		Macro elements (g/kg)		Trace elements (mg/kg)	
Alginic acid (%)	15.60 ± 0.22	N	36.39 ± 0.53	Fe	1091.50 ± 44.73
Amino acids (%)	20.57 ± 0.07	P	3.13 ± 0.21	Mn	23.31 ± 1.15
Fiber (%)	28.68 ± 0.78	K	1.20 ± 0.01	Cu	5.71 ± 0.21
Organic matter (%)	67.00 ± 2.33	Ca	15.43 ± 0.13	Zn	66.88 ± 2.95
		Mg	2.46 ± 0.04	B	46.89 ± 4.85

The main composition of KWE was described by Zheng *et al*.^14^. Values are mean ± SD (n = 3).

**Table 2:** Effects of KWE on germination parameters of pakchoi seeds.

KWE (v/v)	GP (%)	GI	GE (%)	SVI
Control	86.67 ± 2.89 b	6.35 ± 0.17 c	53.33 ± 5.77 bc	460.70 ± 30.74 c
2%	95.00 ± 0.00 a	8.70 ± 0.51 a	71.67 ± 2.89 a	734.67 ± 12.07 a
5%	88.33 ± 2.89 b	7.86 ± 0.11 b	56.67 ± 2.89 b	690.00 ± 18.12 b
10%	71.67 ± 2.89 c	5.42 ± 0.28 d	48.33 ± 2.89 c	264.93 ± 3.93 d
20%	58.33 ± 2.89 d	4.82 ± 0.26 e	46.67 ± 2.89 c	95.73 ± 8.24 e

Values are mean ± SD (n = 100 seeds). The different letter within columns represents the difference is significant at a 5% level with LSD test.
